# Successful gastroduodenal stenting using the endoscopic ultrasound-guided rendezvous technique by Treitz ligament puncture

**DOI:** 10.1055/a-2361-2944

**Published:** 2024-07-29

**Authors:** Shin Yagi, Susumu Hijioka, Kohei Okamoto, Yoshikuni Nagashio, Mark Chatto, Yutaka Saito, Takuji Okusaka

**Affiliations:** 1 68380 Department of Hepatobiliary and Pancreatic Oncology, National Cancer Center Hospital, Tokyo, Japan; 2 37571 Department of Medicine, Makati Medical Center, Manila, Philippines; 3 68380 Endoscopy Division, National Cancer Center Hospital, Tokyo, Japan

Endoscopic gastroduodenal stenting is a widely performed treatment for malignant gastric outlet obstruction (GOO); however, occasionally, the guidewire cannot be passed through the stenosis 1 2 3 . Here, we report a case of successful gastroduodenal stenting using the rendezvous technique with endoscopic ultrasound (EUS) for malignant GOO, which we believe to be the first report of gastroduodenal stenting using the rendezvous technique with EUS.

A 60-year-old woman undergoing chemotherapy for pancreatic cancer presented with vomiting. Computed tomography (CT) showed gastric dilatation and stenosis from the gastric pylorus to the duodenal bulb due to pancreatic cancer ( Fig. 1 ). Endoscopic placement of a gastroduodenal stent was attempted, but the guidewire could not be passed through the stenosis ( Fig. 2 ). Therefore, we decided to puncture the Treitz ligament from the stomach using EUS and place a gastroduodenal stent using the rendezvous technique ( Video 1 ). Using EUS, we continuously delineated the descending part of the duodenum to the Treitz ligament from within the stomach, then punctured the Treitz ligament closest to the gastric wall with a 19-gauge needle, and a guidewire was advanced from the duodenum retrogradely through the stenosis into the stomach ( Fig. 3 ). The scope was then changed to a duodenoscope and a guidewire was advanced from the stomach across the stenosis in an antegrade fashion using the rendezvous technique. Two uncovered gastroduodenal stents were placed in series to cover the stenosis ( Fig. 4 ).

**Fig. 1:**
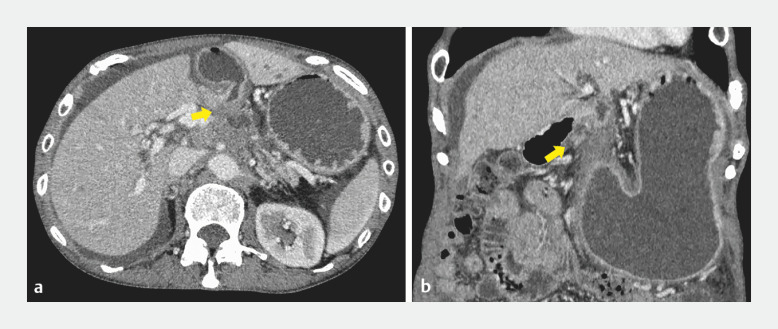
Computed tomography scan images showing gastric dilatation and stenosis (arrow) from the gastric pylorus to the duodenal bulb due to pancreatic cancer on: **a** axial view; **b** coronal view.

**Fig. 2:**
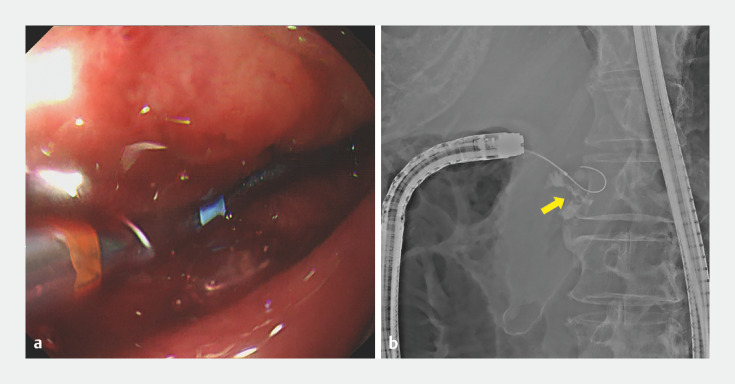
Failed attempts to pass through the stenosis (arrow) using an endoscopic catheter and guidewire are shown on: **a** endoscopic image; **b** radiographic image.

**Fig. 3:**
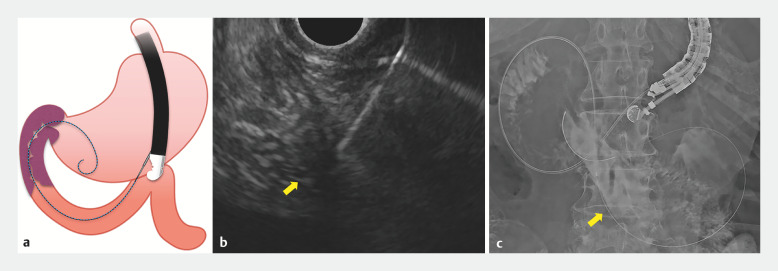
Puncture of the Treitz ligament (arrow) from the stomach is shown on: **a** a schematic image; **b** endoscopic ultrasound image; **c** radiographic image, with the retrograde guidewire (arrow) advanced from the duodenum into the stomach.

**Fig. 4:**
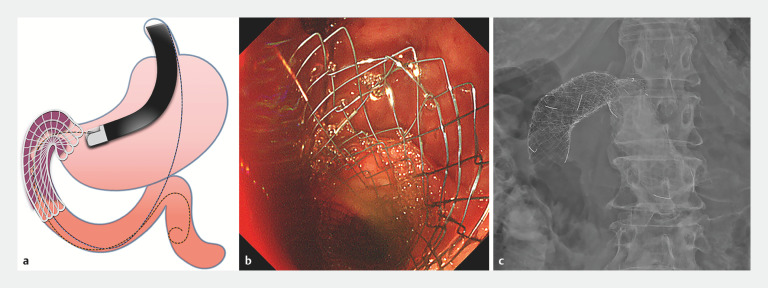
Successful placement of a gastroduodenal stent using the rendezvous technique with endoscopic ultrasound is shown on: **a** a schematic image; **b** endoscopic image; **c** radiographic image.

A gastroduodenal stent is placed using the endoscopic ultrasound-guided rendezvous technique by puncture of the Treitz ligament.Video 1

A CT scan and abdominal radiograph performed the day after the procedure showed no migration of the gastroduodenal stent and resolution of the GOO ( Fig. 5 ). The patient was able to eat and was discharged 5 days after the treatment, with no adverse events.

**Fig. 5:**
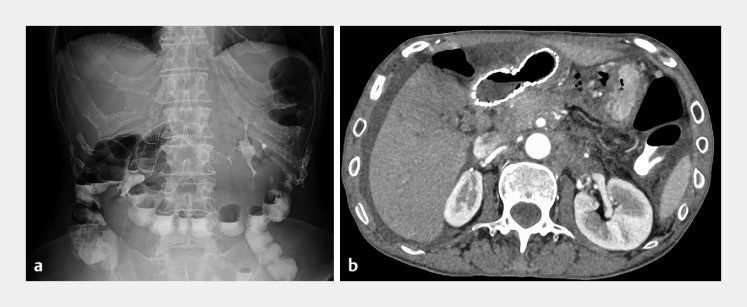
Improvement in the gastric dilatation is seen 1 day after gastroduodenal stent placement on: **a** radiographic image; **b** computed tomography scan image.

In this case, a gastroduodenal stent was successfully placed using the rendezvous technique with EUS. This method may be useful for cases of malignant GOO in which the guidewire cannot be passed through the stenosis.

Endoscopy_UCTN_Code_TTT_1AS_2AI

## Notes

Endoscopy E-Videos https://eref.thieme.de/e-videos
*E-Videos* is an open access online section of the journal *Endoscopy* , reporting on interesting cases and new techniques in gastroenterological endoscopy. All papers include a high-quality video and are published with a Creative Commons CC-BY license. Endoscopy E-Videos qualify for HINARI discounts and waivers and eligibility is automatically checked during the submission process. We grant 100% waivers to articles whose corresponding authors are based in Group A countries and 50% waivers to those who are based in Group B countries as classified by Research4Life (see: https://www.research4life.org/access/eligibility/ ). This section has its own submission website at https://mc.manuscriptcentral.com/e-videos .
